# Assessment of a Wearable Force- and Electromyography Device and Comparison of the Related Signals for Myocontrol

**DOI:** 10.3389/fnbot.2016.00017

**Published:** 2016-11-17

**Authors:** Mathilde Connan, Eduardo Ruiz Ramírez, Bernhard Vodermayer, Claudio Castellini

**Affiliations:** Cognitive Robotics, Institute of Robotics and Mechatronics, German Aerospace Center (DLR)Wessling, Germany

**Keywords:** surface electromyography, force myography, multi-modal intent detection, machine learning, human-machine interfaces, rehabilitation robotics

## Abstract

In the frame of assistive robotics, multi-finger prosthetic hand/wrists have recently appeared, offering an increasing level of dexterity; however, in practice their control is limited to a few hand grips and still unreliable, with the effect that pattern recognition has not yet appeared in the clinical environment. According to the scientific community, one of the keys to improve the situation is multi-modal sensing, i.e., using diverse sensor modalities to interpret the subject's intent and improve the reliability and safety of the control system in daily life activities. In this work, we first describe and test a novel wireless, wearable force- and electromyography device; through an experiment conducted on ten intact subjects, we then compare the obtained signals both qualitatively and quantitatively, highlighting their advantages and disadvantages. Our results indicate that force-myography yields signals which are more stable across time during whenever a pattern is held, than those obtained by electromyography. We speculate that fusion of the two modalities might be advantageous to improve the reliability of myocontrol in the near future.

## 1. Introduction

The human hand is a prodigious natural tool, comprising 27 bones and 33 muscles, resulting in a total of 22 degrees of freedom (DOFs) (Biryukova and Yourovskaya, 1994); its sensorial equipment enables us to drive, browse through the pages of a book, hold and manipulate delicate objects as well as heavy tools. Due to this complexity, artificially reproducing its functions is still a challenge for the roboticians. Nevertheless, a mechatronic tool getting close to the human hand is highly desirable in the context, e.g., of dexterous hand prosthetics. Despite the fact that multi-fingered hand prostheses have appeared on the market during the last decade, their level of abandonment remains relatively high (Biddiss and Chau, 2007; Peerdeman et al., 2011). Touch Bionics' *i-LIMB*, Otto Bock's *Michelangelo*, Vincent Systems' *Vincent Evolution 2* and RSL Steeper's *Bebionic3* are among these examples: with as many as six DOFs, they still are a limited replacement of the hand of an amputee, not mirroring the capabilities of a real human hand. A prosthetic wrist adds at least one DOF to the device and further improves its potential dexterity, but empowering amputees to control such artifacts (myocontrol) is still an open issue.

The academic state-of-the-art of myocontrol relates to the possibility of proportionally and independently controlling each DOF of the prosthesis according to the patient's intent (Sebelius et al., 2005; Cipriani et al., 2011a); however, low stability and accuracy prevent a successful commercialization of such an approach. Myocontrol is still limited to a few DOFs (Arjunan and Kumar, 2010; Yang et al., 2014), and surface electromyography (sEMG) signals are deemed to be no longer enough (Jiang et al., 2012a). Researchers have tried to address this issue by increasing the number of sensors (Tenore et al., 2007), although it is known that four to six channels are acceptable for pattern detection (Young et al., 2012), and/or to find their optimal placement given the characteristics of the stump (Castellini and van der Smagt, 2009; Fang et al., 2015); several pattern recognition algorithms have been studied, such as artificial neural networks (Baspinar et al., 2013), linear discriminant analysis (Khushaba et al., 2009) and non-linear incremental learning (Gijsberts et al., 2014). However, one of the major drawbacks of sEMG signals is their variable nature: sweat, electrode shifts, motion artifacts, ambient noise, cross-talk among deep adjacent muscles and muscular fatigue can crucially affect them (Oskoei and Hu, 2007; Cram and Kasman, 2010; Merletti et al., 2011a; Castellini et al., 2014). In general, any change in the muscle configuration during and after the training of the machine learning algorithm (e.g., the position of the limb and the body and the weights to be lifted during grasping and carrying) must be taken into account (Scheme et al., 2010; Cipriani et al., 2011b). As a result, simultaneous and proportional (s/p) control of each DOF is slow and laborious. Therefore, the application of other types of sensors and sensor combinations is an active field of research (Fougner et al., 2011; Jiang et al., 2012a).

Among non-invasive approaches other than sEMG, electroencephalography (EEG), mechanomyography (MMG), ultrasound imaging also known as sonomyography (SMG), force myography (FMG), functional magnetic resonance imaging and more are considerable options (Lobo-Prat et al., 2014; Ravindra and Castellini, 2014; Fang et al., 2015). Such Human Machine Interfaces (HMIs) have been implemented in distinct studies. However, the research community is recently pushing the development of multi-modal sensing techniques in the field of upper-limb rehabilitation (Fang et al., 2015). For instance, experiments have shown that accelerometer sensor signals can improve the classification accuracy of EMG electrodes (Fougner et al., 2011), as well as a multimodal technique with EMG and Near Infrared Spectroscopy (NIS) (Herrmann and Buchenrieder, 2010), or a combination of EEG and electroneurography (ENG) (Rossini and Rossini, 2010). In brief, a full comparison of the advantages and disadvantages of each type of signal, as well as the possibilities offered by their fusion, is still lacking.

In this study we focus on the joint usage of sEMG and FMG sensors. Surface EMG Merletti et al. (2011b) detects Motor Unit Activation Potentials, that is, electrical fields generated by motor units during muscle contraction, whereas FMG (Phillips and Craelius, 2005; Wininger et al., 2008) detects the pressure exerted by the muscles toward the surface of the skin by volumetric changes induced during muscle activity. Due to the very different nature of the signals gathered by these two techniques, it seems reasonable that they could be proficiently fused in order to better detect a subject's intent. A simple and low-cost option to record FMG signals is represented by force-sensing resistors (FSRs), whose resistance changes according to the pressure applied to them. This is also our option of choice. These sensors are cheap and very compact. Castellini and Ravindra (2014) already proved their effectiveness, and established that finger forces can be predicted with the same accuracy than sEMG sensors (Ravindra and Castellini, 2014). Cho et al. (2016) tested their force sensing system on four amputees and demonstrated that it is possible to classify six primary grips using only FMG with an accuracy of above 70% in the residuum. We describe a novel, modular approach for joint FMG/sEMG intent detection: thanks to a newly developed, fully mobile and wireless acquisition system, we simultaneously gathered FMG and sEMG signals during an experiment in which ten intact subjects performed a repetitive sequence of wrist and hand movements. The collected data was used to assess the desirable characteristics of each modality, while self-assessment questionnaires were used to check that the device was acceptable for the subjects. In the end, we claim that a fusion of the two approaches is potentially better than using them independently for dexterous myocontrol.

## 2. Materials and methods

**Figure 1:**
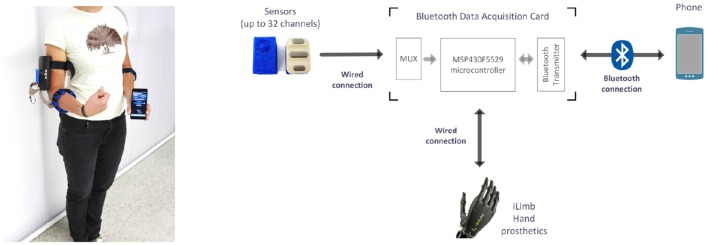
**(Left)** Overview of the experimental setup. The subject is wearing the mobile system with a wired connection to two sensor bracelets, one endowed with FMG sensors, the other one with sEMG sensors. The Bluetooth data acquisition board is located in a box placed on the right upper-arm. **(Right)** Block diagram of the system; an i-LIMB prosthetic hand by Touch Bionics (not used in this experiment) can be directly controlled by the device.

In order to assess the combined data acquisition of sEMG and FMG for myocontrol, we have built a prototype fully wearable, wireless multi-modal myocontrol system. To study its performance, its usability, and the characteristics of the obtained signals, we have involved ten intact human subjects in an experiment with the device as its core. Figure 1 (left panel) shows the system as worn by a subject: the device is composed of three modules: a set of mixed sEMG/FMG sensors (in this case, arranged on two Velcro bracelets), a Bluetooth analog-to-digital conversion board gathering and transmitting the signals, and a smartphone receiving the data via Bluetooth and able to perform myocontrol via a machine learning algorithm. The board was based upon the work of Brunelli et al. (2015), whereas the learning algorithm is Incremental Ridge Regression with Random Fourier Features (see below for more details), already been evaluated (in a non-wearable control system) by Gijsberts et al. (2014) and Strazzulla et al. (2016). Although not extensively used in this specific experiment, the machine learning algorithm can produce control signals in real time and transmit them to the sensor board, which serves as a relay routing them to a hand prosthetic device connected to it. A block diagram of the whole system is presented in Figure 1 (right panel).

### 2.1. Experimental setup

#### 2.1.1. Sensors

Ten Ottobock *MyoBock 13E200* = *50* sensors were used to gather the sEMG signals. They provide on-board amplification, rectification and filtering. Sensors of this kind are a standard in clinical applications, especially in prosthetic sockets.

**Figure 2:**
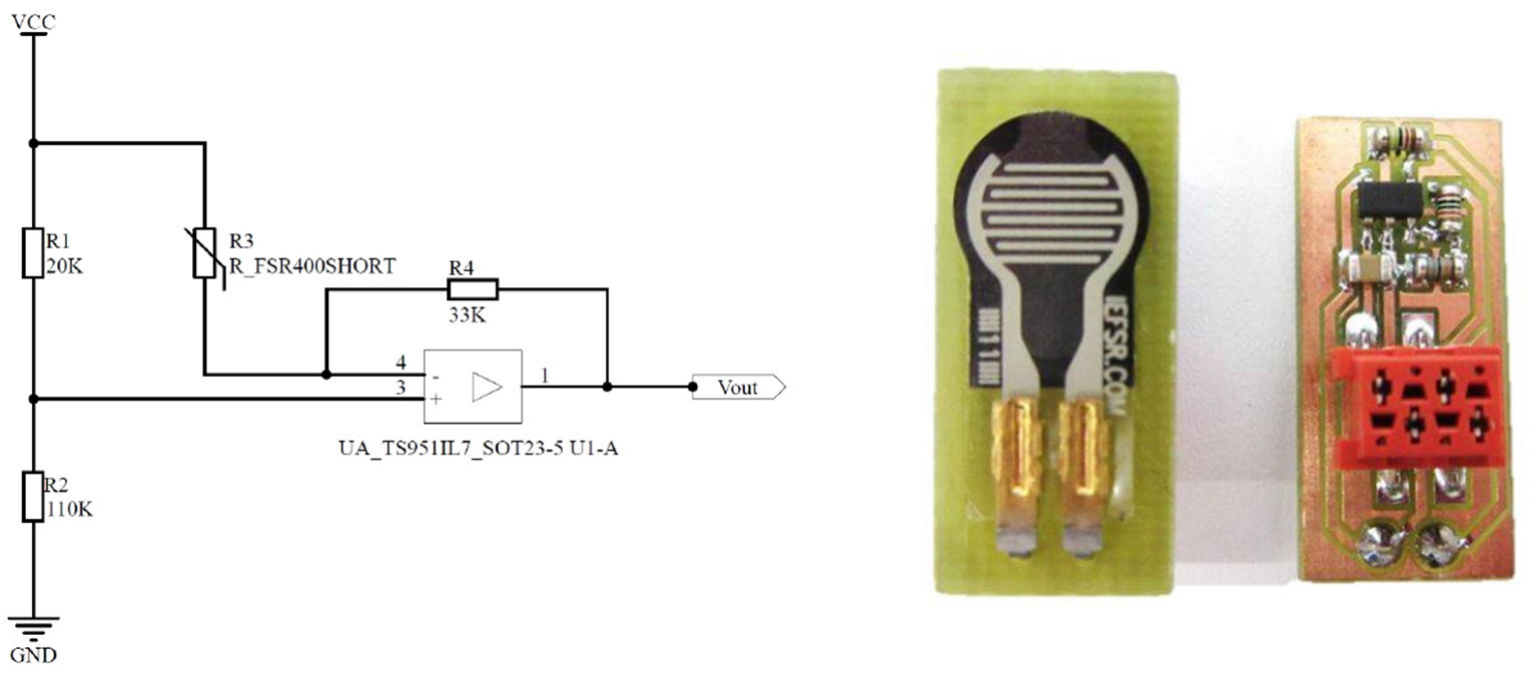
**(Left)** The amplification circuit for the FSR. **(Right)** The FSR sensor's PCB.

FMG signals were registered by ten *FSR 400 Short* force-sensing resistors by Interlink Electronics. Made of a robust polymer thick film, each FSR has a 5.6 mm-diameter sensitive area: when a force is applied to its surface, the electrical resistance of the FSR decreases correspondingly. These sensors are cheap (5€ apiece), but despite the specified remarkably large sensitivity range (0.2N–40N), they have non-negligible hysteresis at high forces, no guarantee of repeatability and a non-linear transfer function. Nonetheless, Castellini and Ravindra (2014) have shown that for small forces (0N–15N) their behavior is largely comparable and their transfer function is almost linear. In our setup, a small printed circuit board with a voltage amplifier (see Figure 2) provides the amplification of the FMG signals. The output of the sensor circuit is $V_{out}=\frac{R_{2}V_{CC}}{R_{1}+R_{2}}-\frac{R_{1}R_{4}V_{CC}}{R_{1}+R_{2}}\times \frac{1}{R_{FSR}}$, yielding a lowest admissible resistance of $R_{FSR}=\frac{R_{1}R_{4}}{R_{2}}=6k\Omega$, which corresponds to a theoretical maximum force observed on the FSR's surface of 3.33*N* (InterlinkElectronics, 2014).

**Figure 3:**
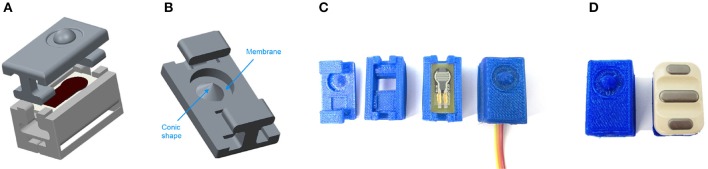
**(A)** Exploded view of the 3D-model of the FSR housing. **(B)** 3D-model of the top part of the FSR housing, where the membrane and the conic shape are identified. **(C)** Assembly process of the housing and the FSR sensor. **(D)** Comparison between the final FSR sensor and the EMG sensor.

In order to provide maximum flexibility in arranging the sensors on the subject's body, specifically on the forearm or stump, uniform 3D-printed housings have been designed for both kinds of sensors. The housings are made of flexible thermoplastic polyurethane and provide adherence to the subject's skin as they are tightened to the arm by a Velcro strap. Each housing provides braces to allow sliding on the strap, so that its position can be individually adjusted and maintained, regardless of the type of sensor (see Figure 3). The FSR sensor housing not only serves as a retainer for the sensor and the amplifier, but furthermore comprises a structured geometric body that is divided into two parts: approximately one half is shaped like a cone and pointing toward the FSR sensor's sensitive area, the other half, shaped like a hemisphere, is pointing toward the skin. This geometric shape has been specifically designed to concentrate the force exerted by the muscles on the FSR sensor's sensitive area. The bearing of this structure is realized by a surrounding, thin and flexible membrane with a thickness of 0.5 mm and a total area of 47.5 mm^2^ (small diameter 6.3 mm, large diameter 10 mm), linking it to the housing. Even if the elastic and damping properties of the membrane have not been subject to further investigation so far, it is assumed that the membrane may increase the signal stability, i.e., it could hypothetically add a mechanical filtering to the bio-signal. This solution offers the capability to create any combination of FMG and sEMG electrodes. In order to gather signals from the complete circumference of the forearm, the sensors are placed evenly spaced around the forearm/stump. This arrangement, also called *low-density surface electrode layout* or *uniform electrode positioning* (Fang et al., 2015), has already been proven effective for robotic hand prosthesis control in a number of previous publications (Castellini and van der Smagt, 2009, 2013).

The typical voltage output of the FSR/amplifier/housing complex has further been characterized. The measurements have been performed with a Zwick Roell *ZMART.PRO* compression test device providing fixture of the sensor setup as well as controlled exertion of force with an accuracy of 100 mN. The corresponding output voltage of the device has been measured with a FLUKE 289 Multimeter with an accuracy of 1 mV. The relationship between voltage output and applied force, in a range from 0 to 5.2 N, is linear with a residual average error of about 7% (see Table 1). It is worth noting that, during the experiment (see below), no FSR ever reached the saturation point, meaning that the FMG signals have all been correctly captured. This matches to a large extent the results obtained in Castellini and Ravindra (2014) and Ravindra and Castellini (2014) for a similar device.

**Table 1:** **Characterization table for one FSR, including the FSR sensor, the amplifier board and the housing**.

**Applied force [N]**	**0.0**	**0.4**	**0.8**	**1.2**	**1.6**	**2.0**	**2.4**	**2.8**	**3.2**	**3.6**	**4.0**	**4.4**	**4.8**	**5.2**
**Output voltage**	**0.00**	**0.16**	**1.02**	**1.37**	**1.66**	**1.98**	**2.31**	**2.51**	**2.73**	**3.10**	**3.27**	**3.49**	**3.72**	**3.85**

*The standard deviation with respect to a linear fit is 6.9%. The provided values have been inverted by the zero-value offset voltage (4.2874V)*.

#### 2.1.2. Analog-digital conversion and data transfer

**Figure 4:**
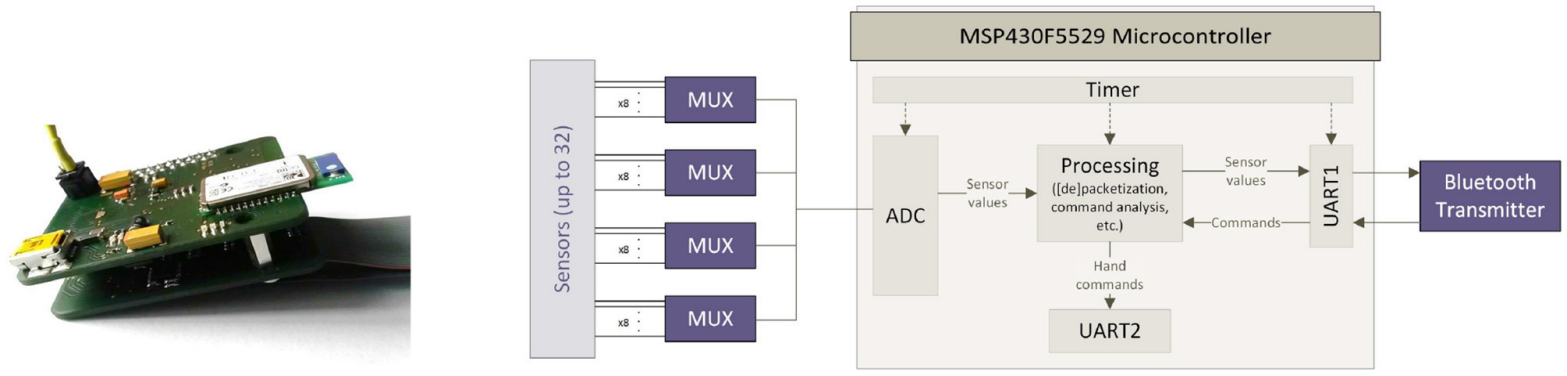
**The bluetooth analog-to-digital conversion board and its functional block representation**.

A Bluetooth ADC board (Figure 4), consisting of a Texas Instrument *MSP430F5529* microcontroller and an on-board Bluetooth chipset, provides analog-to-digital conversion (ADC) of the signals of both the sEMG and FSR sensors, and their wireless transmission. As the microcontroller natively supports only 15 AD-channels, AD-conversion of up to 32 sensors is realized via analog multiplexing, providing a maximum sampling rate of 192.5 Hz for each channel. Since the sEMG sensors already provide rectified and filtered signals with an evaluable bandwidth limited to 10 Hz, the provided sampling frequency is an overshoot (see e.g., Castellini and van der Smagt, 2009). The same argument obviously holds for the FMG signals (Ravindra and Castellini, 2014).

The board employs two UARTs, communicating in turn with the smartphone (via a serial-over-Bluetooth connection) and the prosthesis (in our case, via a simplified RS232 protocol). Hence, the board can also relay control commands to a hand prosthesis. The final cost of the system is estimated to be below 150€.

#### 2.1.3. Myocontrol host

The wearable myocontrol system is completed by a standard commercial smartphone (Huawei *Honor 6* with a commercial value of about 300€), on which the data processing is performed. This device is equipped with a quad-core Cortex-A15 processor running at 1.7 GHz, 2 GB of RAM and a 3100 mAh Li-Po battery, claimed to keep the smartphone running for 2 full days at moderate usage. Its operating system is Android 4.4 KitKat. The smartphone weighs 132 g and easily fits in a pocket (7 × 0.8 × 14 cm) with its 5-inch display. A C# application similar to the one used in Strazzulla et al. (2016) was implemented, optimized and ported to the smartphone. The application enforces the following functionalities: (a) receiving and storing the data from the ADC board's serial-over-BT port; (b) displaying a visual stimulus, both on the smartphone screen and using the prosthesis; (c) building a prediction model for the control commands; and (d) sending them off to the prosthetic hand at a 10 Hz frequency, through the ADC board's serial-over-BT port. The machine learning method of choice was—coherent with our own previous work—Incremental Ridge Regression with Random Fourier Features (Gijsberts et al., 2014). As speculated in previous work and now proven, this method provides real-time capable non-linear multivariate regression while saving a lot of computational resources to the point that the maximum usage of the smartphone's CPU showed to be at 14%. Moreover, with the program running and the cell phone display activated and fully lighted, the battery endurance is at approximately 6 h, whereas an endurance of 11 h can be achieved with the display being switched off. For receiving the sample data and sending control commands to the prosthetic hand, the cell phone's internal Bluetooth peripheral has to be activated all the time.

### 2.2. Experiment description

#### 2.2.1. Subjects

Ten intact subjects, nine of which were right-handed (subject No. 9 being the left-handed one), joined the experiment (3 females and 7 males, 28±7 years old, weighing 72.4±9.91 kg, 177.8±12.14 cm tall). Each subject received a thorough description of the experiment, both in oral and written form. Informed written consent was obtained from all participants. Experiments with sEMG and FMG were approved by the Ethical Committee of the DLR.

#### 2.2.2. Experimental protocol

The experiment consisted of performing ten times the following sequence of wrist and hand movements: (1) wrist flexion, (2) wrist extension, (3) wrist pronation, (4) wrist supination and (5) power grasp. To enforce the opening of the hand the relaxed stance was used, in order to mimic a more natural form of myocontrol. Each movement was visually stimulated on the screen of the smartphone (the name of the required motion would appear on the screen), while the experimenter was visually checking that the movement was actually being enforced, to ensure a correct execution. Each stimulation was administered as follows: the visual stimulus would appear for 2 s to allow the subject reach the full movement, then for 6 s data were captured representing the maximal activation for that particular movement (“activation phase”: only this phase was considered in the offline analysis), then the stimulus would disappear for 2 s to allow the subject return to the resting position. The sequence was administered in the same order to all subjects. The choice of this set of movements was motivated by the well-known importance of controlling at least the wrist pronation/supination (see e.g., Jiang et al., 2012b) together with grasping; for instance, pronation and supination of the wrist are operated by deep muscles (Biryukova and Yourovskaya, 1994), meaning that they are usually hard to detect using sEMG. It is worth mentioning that, to the best of our knowledge, there is so far no commercially available 2-active-DOFs prosthetic wrist, but a few prototypes are being studied [see e.g., the device embedded in the DEKA arm, https://www.youtube.com/watch?v=KCUwoxuAdYQ, and the prototype by Ottobock which appears for instance in Amsuess et al. (2016)].

For this specific experiment, the sensors were separated in two different bracelets, the first one with ten sEMG sensors on the left forearm and the second one with ten FSR sensors on the right forearm. The bracelets were located approximately 10 cm below the subject's elbows. This further choice, rather than that of placing twenty sensors on one single forearm, was motivated by the relatively small space available on the forearm of some of the subjects, which would have potentially limited the adhesion of each sensor to the subjects' skin. (A similar problem was reported of, e.g., in Castellini and Ravindra, 2014). Of course, this diminishes the comparability of the results from the two sets of sensors, but myocontrol literature has already presented cases in which, for instance, training of a machine-learning-based method has been performed bilaterally, i.e., gathering data and training from the intact forearm and predicting using data from the impaired limb (Castellini et al., 2009; Nielsen et al., 2011). In both these works, no difference was reported in performance whether the forearm to be used was the dominant or non-dominant one.

**Figure 5:**
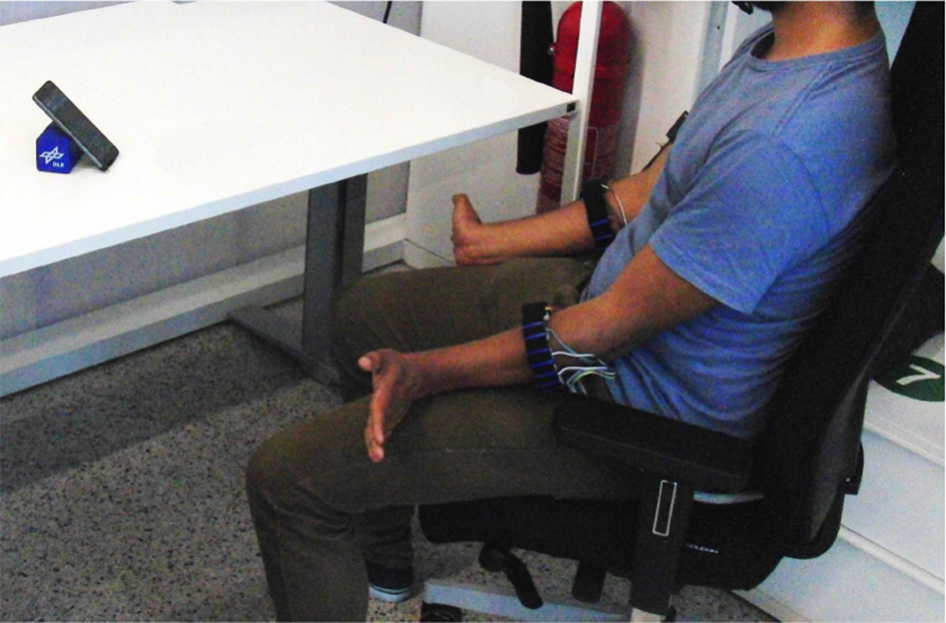
**A bird's-eye view of the experiment**.

During the whole experiment, the subjects sat in a relaxed position with their forearms over their thighs and the hands in a lateral position (with the palms looking toward each other); they were advised to perform each movement bilaterally (Figure 5). The recorded data kept trace of the FSR and sEMG signals as well as of a numerical identifier univocally representing the stimulated movement. This index was used as the ground truth during the supposed maximal activation of the muscles—an instance of *on-off goal-directed stimuli* as already used in, e.g., Sierra González and Castellini (2013).

#### 2.2.3. Data processing

The signals recorded during the experiment were stored to the smartphone's internal memory, then analyzed off-line. Low-pass filtering was applied to both signals (3rd order low-pass digital Butterworth filter with a cutoff frequency of 1 Hz) in order to remove high-frequency disturbances. This is a standard procedure in the field (see e.g., Atzori et al., 2014). For the data processing and the subsequent statistical analysis, the following approaches have been chosen:

1. *Stability over time:* To investigate which type of signal has the most stability over time, the standard deviation of each signal was calculated and Student's paired-sample *t*-test was applied.
2. *Separability of clusters:* Stability of the signals during activation should somehow be reflected in the separability of patterns in the input space, resulting in, e.g., a better classification accuracy when a classification method is employed. Typically (see e.g., Bunderson and Kuiken, 2012), higher separability of clusters means better distinguishability by any pattern classification method and therefore higher stability of the related control. To check whether this was the case, for each subject in the experiment and each pair of clusters (*C*_*i*_, *C*_*j*_) we evaluated Fisher's Separateness Index (Fisher, 1936), defined as the maximum value over *w* of *J*(*w*), where $J\left(w\right)=\frac{w^{T}S_{B}w}{w^{T}S_{W}w}$. Here *S*_*B*_ is the between-clusters scatter matrix, while *S*_*W*_ is the within-clusters scatter matrix. *S*_*B*_ is given by $S_{B}\;=\;\left(\mu_{i}-\mu_{j}\right){\left(\mu_{i}-\mu_{j}\right)}^{T}$ where μ_*i*_, μ_*j*_ are the means of clusters *C*_*i*_, *C*_*j*_, while $S_{W}=\underset{n=i,j}{\sum }\underset{x\in clust_{n}}{\sum }\left(x-\mu_{n}\right){\left(x-\mu_{n}\right)}^{T}$, where *x* are the samples in each cluster. Each pairwise Fisher's index was averaged across all subjects and collected in a matrix *S* = {*s*_*ij*_}.
3. *sEMG/FMG regression for myocontrol:* A comparative regression accuracy analysis was performed, in order to assess whether sEMG, FMG or their juxtaposition would be significantly better in the framework of wrist/hand prostheses control. The learning algorithm of choice was Incremental Ridge Regression with Random Fourier Features, already successfully used multiple times, e.g., in Gijsberts et al. (2014) and Ravindra and Castellini (2014). Ridge Regression builds a linear model *f*(*x*) = *w*^*T*^*x*, where *x* denotes the sensor values, *w* is a weighting vector and *f*(*x*) is the predicted output; Random Fourier Features further employ a non-linear mapping from the input space to a higher-, finite-dimensional feature space, where the linear regression is more likely to succeed. (For more details about this algorithm applied for hand prosthesis control, see Gijsberts et al. (2014)). Ten-fold “leave-one-repetition-out” cross-validation was applied by training each machine on nine of the ten repetitions and testing on the remaining one. The input space was chosen to be either the FMG values, the sEMG values, or their combination, meaning that the FMG and sEMG samples were simply stacked in a 20-dimensional vector and used with the same learning method. The prediction accuracy was measured using the normalized Root Mean-Squared-Error (nRMSE) between the predicted values and the stimulus values. A one-way ANOVA test was performed to investigate whether there was a statistically significant difference in between the three inputs.

#### 2.2.4. Satisfaction surveys

Additionally, at the end of the experiment, three surveys were administered to each subject, in order to complete the envisaged system assessment with respect to its usability: the System Usability Scale (SUS) (Brooke, 1996), the NASA Task Load Index (NASA, 1986) and a reworking of the Microsoft Desirability Toolkit by Travis (2008). The SUS consists of ten questions (Table 2) with answers represented on a 5 point Likert scale (1 - *strongly disagree* to 5 - *strongly agree*). The scoring in this survey is such that the answers to the *strongly agree* positive questions and to the *strongly disagree* negative questions generate a higher impact over the final score. The NASA Task Load Index provides an overall workload score on six subscales: Mental, Physical and Temporal Demands; Own Performance, Effort and Frustration. For each subscale, the answer could be in a range of 21 points, reaching from *very low* to *very high* (Table 2).

**Table 2:** **The statements found in the SUS and NASA TLX surveys**.

**SUS SURVEY**
I felt comfortable with the device.
I found the device unnecessarily complex.
I thought the device was easy to use.
I think that I would need the support of a technical person to be able to use this device.
I found the various functions in this system were well integrated.
I thought there was too much inconsistency in this device.
I would imagine that most users would learn to use this device very quickly.
I found the device very cumbersome to use.
I felt very confident using the device.
I needed to learn a lot of things before I could get going with this device.
**NASA TLX SURVEY**
How mentally demanding was the task?
How physically demanding was the task?
How hurried or rushed was the pace of the task?
How successful were you in accomplishing what you were asked to do?
How hard did you have to work to accomplish your level of performance?
How insecure, discouraged, irritated, stressed, and annoyed were you?

Lastly, Travis's survey consists in a series of “reaction cards” with adjectives that could be applied to the system to be tested; the user is asked to select the five cards that most closely match their personal reactions to the system. For the experiment, we used a list of 75 adjectives instead of the cards (most of them based on Travis's questionnaire), then the subject was asked to choose all adjectives he or she felt more related with the device. After that, in a more precise selection, the user had to choose only the 5 most important words and try to give a simple reason about his or her decision.

## 3. Experimental results

### 3.1. Stability over time

**Figure 6:**
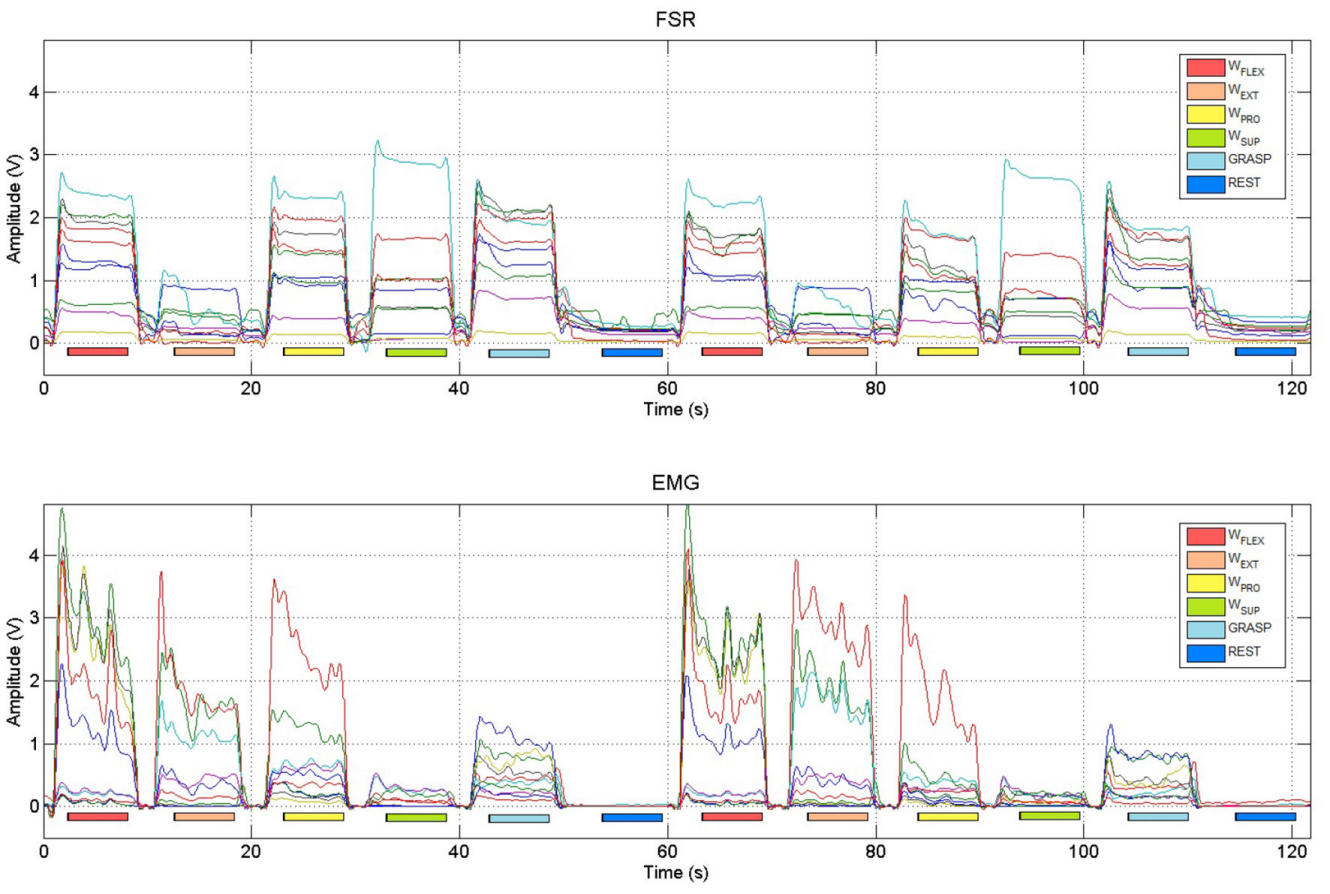
**Typical FSR and sEMG signals obtained from two repetitions of the instructed hand and wrist movements (wrist flexion, wrist extension, wrist pronation, wrist supination, power grasp and rest)**. Colored bars denote the activation phases, during which data were collected to represent the maximal activation of the stimulated movement.

Figure 6 shows typical FMG and sEMG signals obtained while a subject was performing two repetitions of the five instructed movements (plus the resting state). Due to the carefully chosen amplification/filtering stages of the sensors themselves, the amplitude of the signals obtained from both FMG and sEMG sensors are comparable, and each single produced movement appears as a distinct pattern, well separated in time from the next one as well as from the 2-s intervals allowed for resting and for preparing the next movement. Such behavior is clear, e.g., during the wrist-flexion movement enforced from 60 to 70 s in the Figure (second red bar just above the *x*-axis).

Visual inspection seems to indicate that FMG signals are more stable over time *while the subjects are holding the position* than sEMG signals: this is apparent by looking at the “plateaus” created by the FSRs while each movement was enforced; as opposed to that, sEMG signals exhibit the typical oscillating down-ramp pattern due to muscular motor-unit recruitment (Merletti et al., 2011a,b). To verify that this is the case in general, we evaluated the standard deviation of the FMG and sEMG signals obtained by each subject while performing the first three repetitions of the wrist flexion movement considering the signals during the activation phases only. (Only the three sensors for each set that exhibited the highest amplitude were taken into account.) Considering Table 3, sEMG signals actually exhibit a significantly higher standard deviation when compared to FMG signals (mean values 0.0087 and 0.0025 in turn, Student's paired-sample *t*-test *p* < 0.01).

**Table 3:** **Standard deviation of FMG and sEMG sensor signals obtained by each subject during the first three repetitions of the wrist flexion movement**.

**Subject #**	**1**	**2**	**3**	**4**	**5**	**6**	**7**	**8**	**9**	**10**	**Mean**
FMG	0.0018	0.0026	0.0006	0.0030	0.0033	0.0042	0.0018	0.0016	0.0028	0.0031	0.0025
sEMG	0.0202	0.0111	0.0019	0.0038	0.0144	0.0082	0.0037	0.0065	0.0082	0.0091	0.0087

*Considering the three sensors that exhibited the highest signal amplitude*.

### 3.2. Separability of clusters

**Figure 7:**
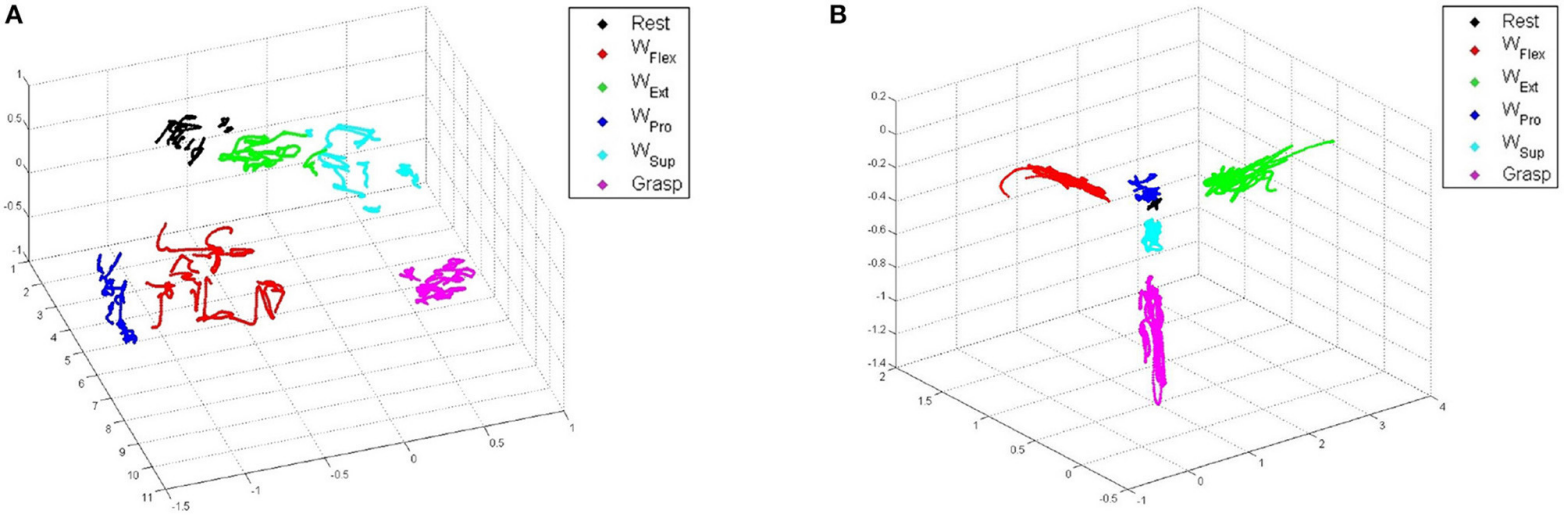
**3D-reduced PCA projections of typical data for each type of sensor, colored according to the stimulated movements**. **(A)** FMG. **(B)** sEMG.

Figure 7 shows typical FMG and sEMG data reduced to three dimensions via Principal Component Analysis (PCA) and colored according to each movement for the cluster separability analysis carried out for both input spaces. In the figure, sEMG clusters appear more stretched than FMG clusters, a behavior very likely due to the above-mentioned oscillations while a movement is being held.

**Figure 8:**
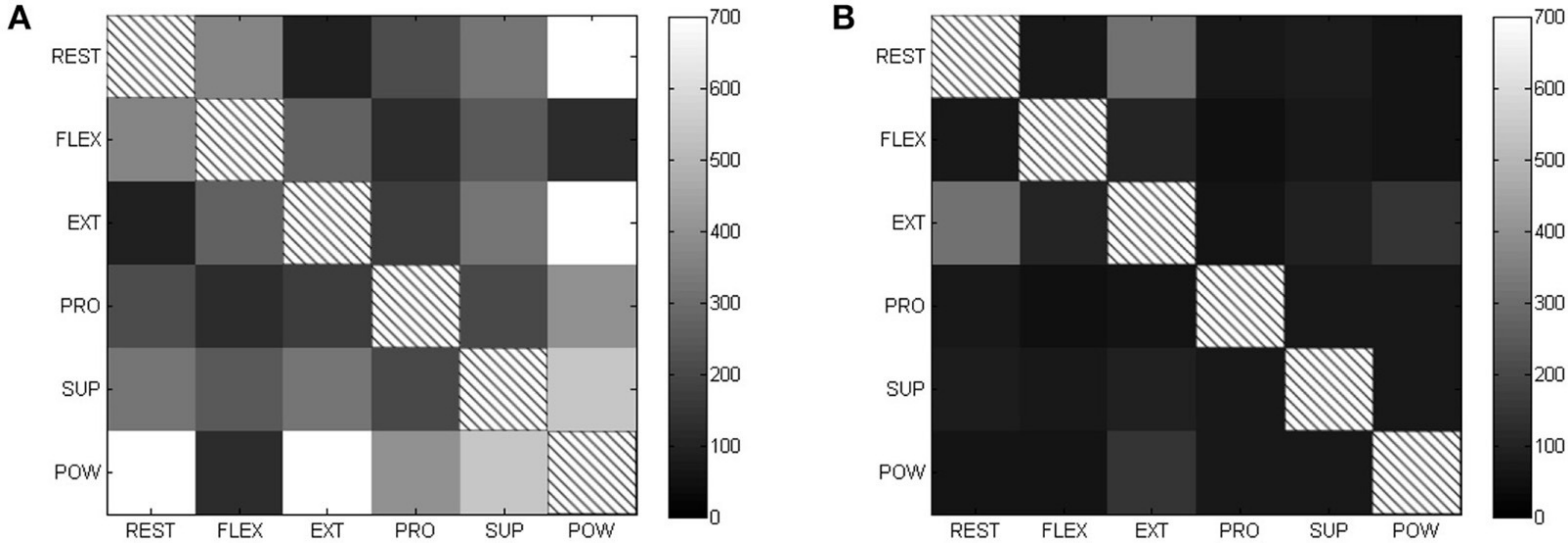
**Fisher's Index matrices for each type of sensor (higher is better)**. **(A)** FSR Fisher's index. **(B)** EMG Fisher's index.

Figure 8 shows the matrices for sEMG and FMG, while Table 4 lists the means of Fisher's Indexes for each subject (the diagonal-zero values are not considered to evaluate the mean values). The Fisher's Index of FMG is higher (therefore better) than that of sEMG (mean values 368.82 and 94.1) with high statistical significance (Student's paired-sample *t*-test *p* < 0.001).

**Table 4:** **Mean Fisher's Index values for each subject and type of sensors**.

**Subject #**	**1**	**2**	**3**	**4**	**5**	**6**	**7**	**8**	**9**	**10**	**Mean**
Fisher's Index, FMG	291.08±227.9	616.47±893.5	340.22±259.7	108.11±50	157.50±72.8	391.26±527.2	432.3±342.2	513.18±453.9	455.85±532.4	382.21±722.2	368.82±154.2
Fisher's Index, sEMG	87±74.3	69.46±45.2	71.24±23.6	38.03±16.9	208.34±279.3	67.78±23.3	66.12±46.2	25.69±11.3	239.38±387.1	67.93±39.1	94.1±71

### 3.3. sEMG/FMG regression for mycontrol

**Figure 9:**
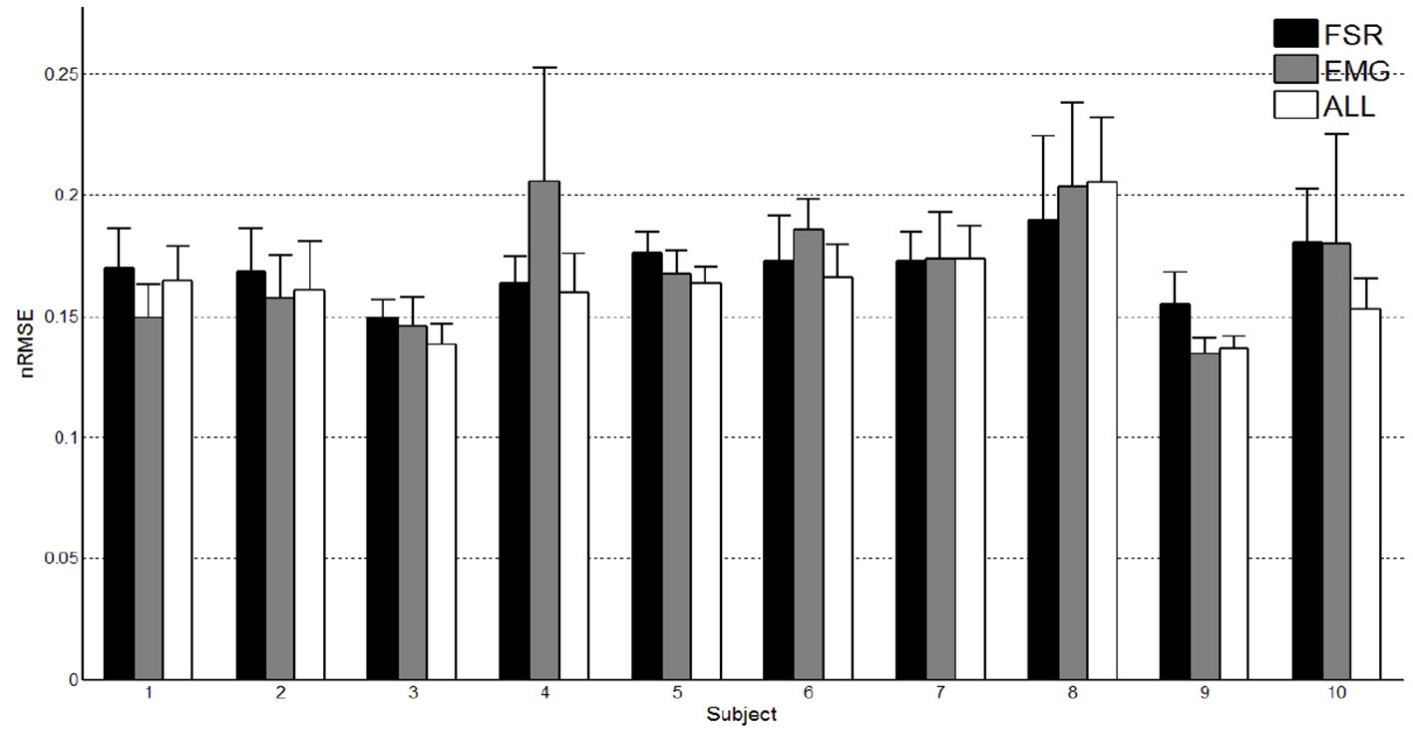
**Prediction accuracy obtained by FMG, sEMG and their combination**.

Table 5 shows the prediction accuracy obtained by each subject on one movement repetition (the nRMSE value showed is a mean of all the nRMSE obtained by the cross-validation). Figure 9 shows the nRMSE values for all subjects. While the nRMSE values range from 0.13 to 0.21, in line with previous literature (Ravindra and Castellini, 2014), no statistically significant difference in accuracy is apparent (one-way ANOVA *p* > 0.05).

**Table 5:** **Prediction accuracy (nRMSE) obtained by Incremental Ridge Regression with Random Fourier Features when trained on FMG, sEMG and combined values**.

**Subject #**	**1**	**2**	**3**	**4**	**5**	**6**	**7**	**8**	**9**	**10**
FMG	0.17±0.016	0.1687±0.0173	0.1495±0.007	0.1636±0.011	0.1762±0.0087	0.173±0.0184	0.1728±0.0121	0.1893±0.0352	0.1548±0.0133	0.1803±0.0222
sEMG	0.1494±0.0138	0.1573±0.0175	0.1458±0.0123	0.2056±0.047	0.1673±0.0098	0.1857±0.0126	0.1736±0.0193	0.2037±0.0343	0.1346±0.0061	0.1801±0.045
Comb.	0.1649±0.0137	0.1608±0.0199	0.1385±0.0083	0.1595±0.0161	0.1636±0.0066	0.1658±0.0139	0.1736±0.0139	0.2054±0.0267	0.1367±0.0048	0.1526±0.013

### 3.4. User satisfaction

For the SUS, the total result of each subject and the mean score are presented in Table 6. Notice that the higher the score, the more usable the user judged the device. In this survey, the statements that had the worst scores were “I think that I would need the support of a technical person to be able to use this device” and “I felt very confident using the device.”

**Table 6:** **Subject's system usability total scores**.

**ID**	**1**	**2**	**3**	**4**	**5**	**6**	**7**	**8**	**9**	**10**	**Mean**
SUS	85	90	100	77.5	92.5	67.5	75	85	100	75	84.75

**Figure 10:**
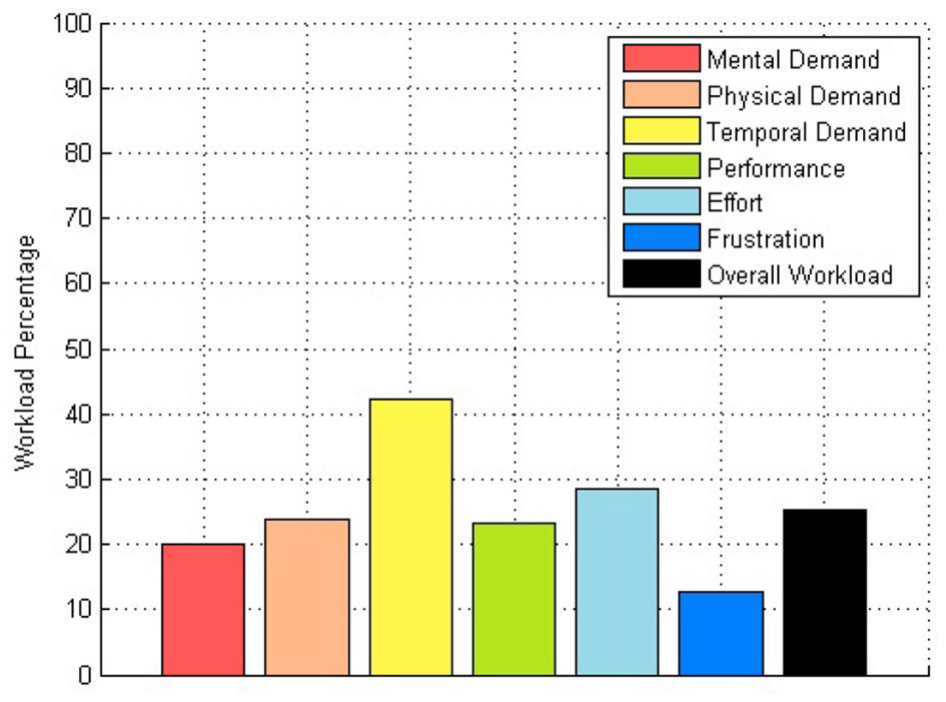
**Workload percentage plot**.

For the NASA TLX, the total result for each subscale of each subject and overall workload are shown in Table 7. Here the highest the score, the more workload the user had, when using the device. A plot with average percentages of the workload by subscale is visible in Figure 10.

**Table 7:** **NASA TLX workload percentages**.

**ID**	**1**	**2**	**3**	**4**	**5**	**6**	**7**	**8**	**9**	**10**	**Mean**
Mental demand	66.66	19.04	9.52	14.28	23.8	28.57	9.52	14.28	4.76	9.52	20
Physical demand	33.33	23.8	14.28	14.28	23.8	23.8	9.52	14.28	4.76	76.19	23.8
Temporal demand	71.42	61.9	52.38	14.28	52.38	57.14	14.28	61.9	4.76	33.33	42.38
Performance	28.57	28.57	19.04	14.28	14.28	19.04	52.38	14.28	4.76	38.09	23.33
Effort	52.38	19.04	23.8	9.52	52.38	19.04	52.38	14.28	4.76	38.09	28.57
Frustration	19.04	14.28	4.76	19.04	23.8	9.52	9.52	9.52	4.76	14.28	12.85
Overall WL	45.2381	27.77	20.63	14.28	31.74	26.19	24.6	21.42	4.76	34.92	25.15

(Subject #9 actually gave a uniform scoring.)

The first two surveys applied comprised suitable results with a usability score of almost 85% and an overall workload of 25%.

**Figure 11:**
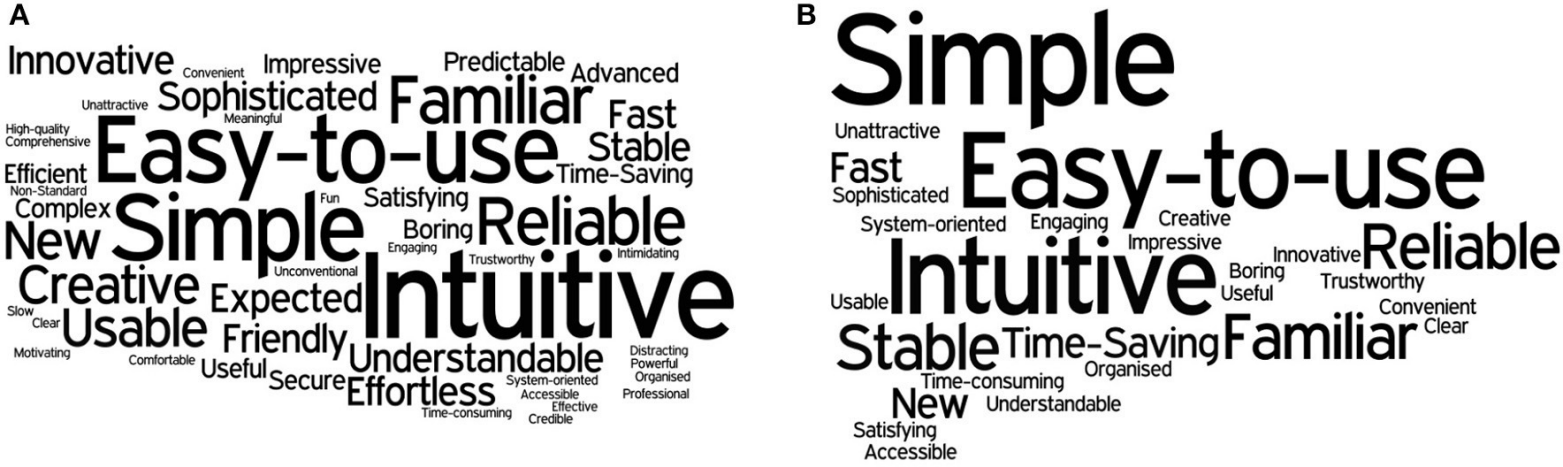
**Word clouds from the results of the desirability survey**. **(A)** First selection word cloud. **(B)** Final 5 words selection cloud.

Finally, for the desirability survey proposed by Travis, two kinds of results were obtained, the first one using all the words chosen by the user in the first selection, and the second one considering only the 5 final selections. In order to have a different visualization, two word clouds have been created (Figure 11), where the bigger and darker the font is, the more often the word was selected.

Considering only the most common adjectives in the final selection, the device can be considered *Simple, Intuitive, Easy to use, Familiar, Reliable* and *Stable*. An important thing to mention about this last survey is that even though the instructions and the questions were oriented toward the device, some answers referred to the experiment performance. For instance, the adjective *familiar* was chosen in some cases because the subject had already performed other experiments with EMG sensors before taking part in this specific experiment.

**Figure 12:**
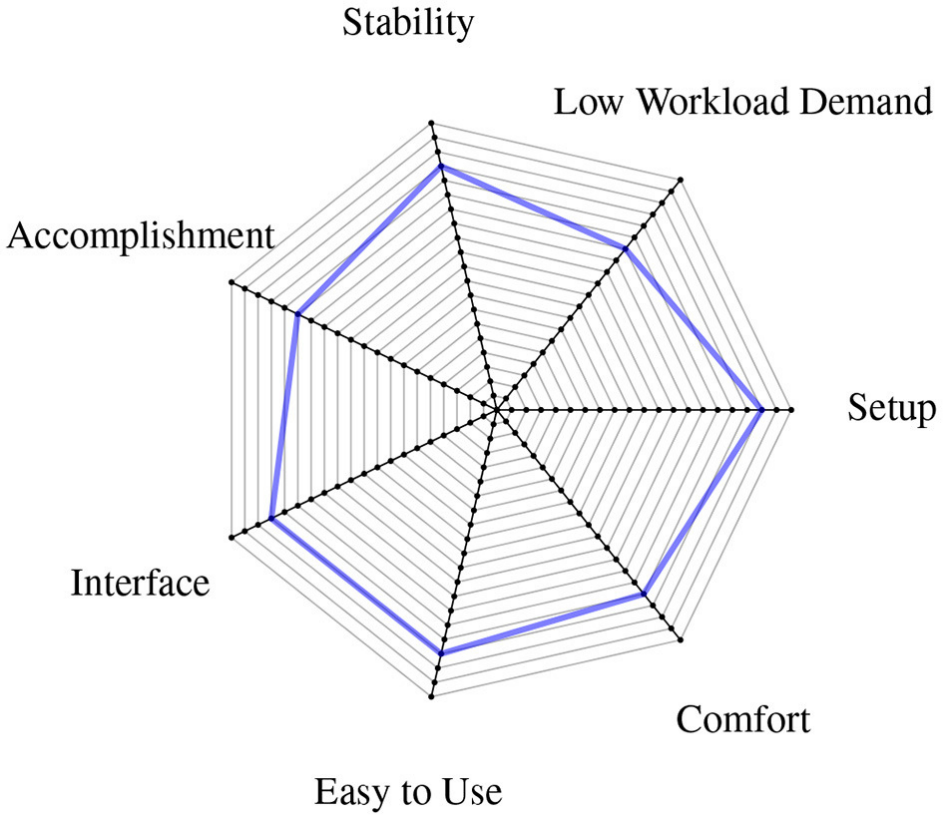
**Radial chart showing the user satisfaction surveys' results summarized in different device features**.

To highlight the results of the user satisfaction surveys, a radial chart (Figure 12) was built, which separates the results in different categories (Low Workload Demand, Stability, Task Accomplishment, Interface, Easy to Use, Comfort and Setup) and represents the main features appreciated by the user. The figure for Low Workload Demand has been inverted on the scale to achieve better comparability with the other figures (i.e., Workload Demand is positively rated if being low). All of these categories can furthermore be separated in two kinds of classes: usability related features (Interface, Easy to Use, Comfort and Setup) and performance related features (Workload Demand, Stability and Task Accomplishment).

## 4. Discussion

This work had two main aims: to assess whether a wearable combined sEMG/FMG device would be accepted by human subjects using it, and to determine whether significant qualitative and quantitative differences could be observed between sEMG and FMG signals obtained during a simple experiment aimed at myocontrol.

### 4.1. Acceptance of the device

Consider Section 3.4, in particular Figure 12. The results clearly show that the device, together with its user interface, posed no problems to the subjects using it, and even had some appeal. Even though the subjects involved in the experiment are not part of the potential user population (i.e., amputated subjects controlling a self-powered prosthesis), their opinions and impressions about the device are helpful for future improvements and corrections. The subjects uniformly reported that the device felt reliable and stable; that the setup was easy to use, simple and comfortable, with a low frustration rate; that the user interface was friendly, intuitive and well structured; and that the data acquisition still required a considerable amount of time (this aspect having the highest workload demand).

The keywords obtained in Travis's survey, the workloads obtained in the NASA TLX survey, and the successful performance reflected in the SUS test, already give hints about the usage risk of a possible future medical device. Of course, this cannot replace a thorough risk analysis and a widespread usability study. Furthermore, it must be remarked that the positive results obtained in the surveys could have been influenced by the subjects' behaviors themselves, as explained by Travis. All in all, it is worthwhile to stress that no online experiment was performed in this study, therefore, from the user survey results, we can provide no conclusive results about either the performance of this device in general, or the usefulness of a combined sEMG/FMG approach in myocontrol.

### 4.2. Comparison of sEMG and FMG

In previous literature (Yungher et al., 2011; Ravindra and Castellini, 2014) it has been shown that FMG shows higher *overall* stability over time than sEMG, meaning that, e.g., the variance of its signals is lower than that of sEMG, while human subjects are engaged in repetitive, fatiguing tasks. This is probably due to the lower influence that muscle fatigue has on FMG signals, due to its nature. Now, from the qualitative/quantitative comparison of sEMG and FMG carried out in Sections 3.1 and 3.2, two statistically significant differences between the two approaches emerge, namely (a) that FMG signals are more stable over time *during single movements too*, and (b) that they generate better separated patterns in the input space. Most likely, (b) is a consequence of (a); we speculate that this difference might arise from the very nature of the sEMG signals, which exhibit noise due to the recruitment of motor units while keeping an isometric hand/wrist posture. Of course, FMG would not be affected by this problem. All things considered, it seems reasonable to claim that FMG signals are more stable than sEMG ones.

About pattern separateness: if a classification approach were to be used to enforce myocontrol using such signals, better pattern separability would definitely represent a further advantage of FMG with respect to sEMG; in the case of simultaneous and proportional control, however, it is not clear whether this is an advantage or not. This kind of control requires some way to understand not only what pattern the subject desires, but also how much force/torque is involved; distant and smaller clusters for the maximal activations might contain less information about this specific feature. In our case, the regression method used in Section 3.3 is trained on maximal and minimal activation signals only, whereas it predicts the intermediate activation values by non-linear interpolation. We are in no position at this time, to claim that FMG or sEMG is better in this case (and this is reflected in the non-stastically-significant accuracy results obtained by such method, see Table 5 again).

On a more qualitative side, we note that FMG, at least enforced using this cheap approach (that is, Force-Sensing Resistors), presents the drawback of being affected by hysteresis. Although the return to the resting state is apparent, this induces the FMG signals even to rise during the resting states, but not to come back exactly to zero or the previously measured resting states after a movement is performed. As opposed to this, sEMG signals remain almost at zero even when the user is not in the same initial position; this seems reasonable, since the resting phase involves no muscle activation. This problem can be countered by employing a smarter technique to gather FMG signals, for instance the capacitive approach, or (high-density) tactile sensing. Also, a fully-fledged, online FMG approach will need to take into account the inevitable artifacts generated by the arm/forearm movement (i.e., accelerations inducing pressure on the sensors) and those induced by touching the socket, bumping into objects, laying the stump on a table, etc.

What we can conclude, we believe beyond any reasonable doubt, is that sEMG and FMG can be miniaturized and employed in such a framework, and that they carry different kinds of information, leading to different behaviors and signal features. We speculate that a structured sensor-fusion approach (that is, deeper than simply stacking the signals as we have done in this work) might lead to a better exploitation of each modality's characteristics.

One last remark is in order about the cost of each approach. Apparently, FMG is up to two orders of magnitude cheaper than sEMG, but this is mainly due at this stage to (a) it being enforced through Force-Sensing Resistors, which might not live up to the expectations as previously remarked; and (b) the necessity, in the very end, to produce a *medically certified* FMG approach and device, which might dramatically raise its costs. Again, we believe that an *integrated* approach is the way ahead to improve myocontrol using this still novel technique alongside sEMG. To this aim, the assessment of the wearable sEMG/FMG device we carried out is promising, as it shows that at least the required electronic machinery can be embedded in an effective, light and acceptable device.

### 4.3. Conclusions and future work

In this article, we have described a wearable, integrated sEMG/FMG system, targeting myocontrol and human intent detection. The experiment we conducted endorses the system's high degree of usability, indicating that it has the potential to become an integrated medical product. Still, the autonomous mobility of about 11 h is restricted by the cell phone battery, thus recharging strategies during the patient's daily use or the deployment of a secondary device could be a solution for now. In near future, the development of a highly integrated, low energy system, where the phone serves only for teaching and displaying information, seems to be advisable. Additionally, we explored the application of FMG as a potential complement to sEMG and provided evidence that the two techniques can be integrated, but that a smart sensor fusion approach might be required to obtain the best results. Results from user satisfaction surveys presented in the paper give strong indication for the setup to be on the right track. In the near future, more experiments are planned to check the feasibility of mixed sEMG/FMG for online myocontrol, possibly down to the level of individual finger movements, and immersed in daily-life activities.

## Author contributions

System Design BV, MC, CC; Software Design MC, BV; Test setup and conduction of user Surveys ER, MC, CC; Analysis and Interpretation of data ER, CC; Drafting of manuscript MC, ER, BV; Internal Revision BV, CC; Critical Revision BV, CC, MC, ER; Final Approval MC, ER, BV, CC; Agreement on accountability by MC, ER, BC, CC.

### Conflict of interest statement

The authors declare that the research was conducted in the absence of any commercial or financial relationships that could be construed as a potential conflict of interest.
